# EuroQol Toddler and Infant Populations (EQ-TIPS): Age-Related Differences in Performance

**DOI:** 10.3390/children11081034

**Published:** 2024-08-22

**Authors:** Janine Verstraete, Razia Amien, Lasse Herdien

**Affiliations:** 1 Department of Paediatrics and Child Health, University of Cape Town, Cape Town 7925, South Africa; 2 Department of Health and Rehabilitation Sciences, Division of Physiotherapy, University of Cape Town, Cape Town 7925, South Africa; razia.amien@alumni.uct.ac.za

**Keywords:** child, infant, toddler, preschooler, Health-Related Quality of Life, HRQoL, proxy, outcome measure, EQ-5D, EQ-TIPS

## Abstract

Objectives: The EQ-TIPS was developed to measure the Health-Related Quality of Life in infants/toddlers. Considering the rapid development in this period, this study aimed to investigate age-related variations in EQ-TIPS performance. Methods: Data from 551 infants/toddlers living with a health condition were analysed. Infants/toddlers were grouped by age: 0–6 months (n = 100), 6–12 months (n = 95), 12–24 months (n = 147), and 36–48 months (n = 97). Differences in item responses and item correlations across age groups were calculated by Kruskal–Wallis and Spearman’s correlations, respectively. Results: The report of problems was significantly higher for movement, play, and communication in the 36–48-month group compared to the 0–6-month group. There were strong correlations (r > 0.50) across all age groups between play and movement and communication and social interaction/play; neither pain nor eating showed a clear pattern of association. Conclusions: There is an age-related difference in the reporting of items linked to developmental milestones (movement, play, and communication) with most problems reported in the 36–48-month group when deviation from peers and continued dependence on caregivers is notable. Consideration should be given to including broader examples of play in the EQ-TIPS. Redefining the items to represent social communication and/or (social) emotion, rather than communication and social interaction, may be warranted. Future research should explore the psychometric performance of items to further inform item inclusion and/or revision.

## 1. Introduction

Early childhood development, particularly the period of infancy and toddlerhood, is widely recognised as the most important phase in the human lifespan due to the rapid development of the central nervous system [1]. Experiences during this early period exert major and often enduring effects on the course of brain structure and function, strongly influencing wellbeing, nutritional status, mental health, heart disease, competence in literacy and numeracy, criminality, and economic participation in the future [1]. Thus, experience of poor health and adverse environmental exposures need to be managed actively and well. The measurement of developmental outcomes and Health-Related Quality of Life (HRQoL) is therefore becoming increasingly important in this young age group [1,2,3,4,5].

The EuroQol measures for adults (EQ-5D-3L and EQ-5D-5L) and youth (EQ-5D-Y-3L and EQ-5D-Y-5L) are frequently used to measure HRQoL from 4 years old into adulthood (18+ years) [6]. The EuroQol Toddler and Infant Populations (EQ-TIPS), formerly the TANDI, is an experimental measure developed by the EuroQol Group to measure and value HRQoL in infants and toddlers [7]. The EQ-TIPS uses the same format as the EQ-5D-Y and other EQ tools and consists of two pages: the descriptive system and the EQ visual analogue scale (EQ VAS). The EQ-TIPS, once an official instrument, will allow for the measurement and valuation of HRQoL with EQ tools across the lifespan. EuroQol experimental measures are tools which are still under development and may change as development proceeds [8]. As such, the EQ-TIPS descriptive system is likely to be refined following results from ongoing international research.

To date, EQ-TIPS evidence has been generated in South Africa on the English [9,10,11], Afrikaans [12], and isiXhosa [13] versions. Although the EQ-TIPS was originally developed to measure HRQoL in children aged 0–3 years, there has been a suggestion to extended this to 4 years based on evidence that it performs better than the EQ-5D-Y-3L in this age group [10]. The proposed age range for the EQ-TIPS is, however, wide when one considers other generic, multi-attribute utility instruments for infants and/or toddlers [3], e.g., Infant Health-Related Quality of Life Instrument (IQI) (0–12 months) [14], PedsQL for Infant (1–12 months and 13–24 months) [15] and Toddlers (2–4 years) [16], and the Health Utilities Pre-School Children (HuPS) (2–4 years), previously the Health Status Classification System for Pre-School Children (HSCS-PS) [17,18].

Given the important potential future uses of the EQ-TIPS for the measurement of HRQoL in infants/toddlers and its potential to inform lifetime models, its ability to sensitively and accurately capture HRQoL across the age range is imperative. During the development process, great consideration was given to whether one (applicable to the entire age range of 0–3 years) or multiple measures (applicable to narrower age groups) were needed [9]. Although previous South African studies of the EQ-TIPS [9,12,13] have explored age-related performance, the sample per age group has been modest, and further investigation is warranted to inform the future development of the descriptive system. Similarly to the EQ-TIPS, the PedsQL, IQI, and HuPS are relatively newly developed, with a resultant dearth of evidence to support their psychometric performance by age [3,4]. To the best of the authors’ knowledge, there are no studies comparing the age-related performance for the PedsQL, IQI, or HuPS. The aim of this study was, thus, to investigate age-related variations in EQ-TIPS performance with pooled data from previous studies conducted in South Africa.

## 2. Methods

### 2.1. Participants

Data from previously published EQ-TIPS studies in South Africa were pooled for analyses [9,10,11,12,13]. Only data from infants and toddlers aged between 0 and 48 months with a health condition were included. All previously collected data from children with a health condition were from the same tertiary paediatric hospital located in Cape Town, South Africa.

### 2.2. Measures

The EQ-TIPS is a proxy report instrument intended for completion, in most cases, by the parent or primary caregiver of infants and toddlers. The EQ-TIPS descriptive system currently comprises the following six items: movement, play, pain, social interaction, communication, and eating. Each item has three levels of severity corresponding to no problems, some problems, and a lot of problems. Verstraete et al. has previously published a sample of the descriptive system, previously referred to as the TANDI [9].

The proxy respondent, typically the parent, is asked to indicate the young child’s health state on the day of questionnaire completion by ticking the box next to the most appropriate statement in each of the six items. The response for each item is defined as a 1-digit number that expresses the level selected for that item, where ‘no problems’ is assigned a ‘1’ and the most extreme level a ‘3’. For example, the EQ-TIPS health state 111223 describes someone with no problems with movement, no problems with play, no problems with pain, some problems with social interaction, some problems with communication, and a lot of problems with eating. The best health state described by the instrument is coded as 111111, describing ‘no problems’ in each of the dimensions. Thus, the EQ-TIPS has 729 (36) unique health states.

In the future, it is likely that each EQ-TIPS health state will be associated with a societal preference weight (or ‘utility’), which will be derived from an EQ-TIPS value set and will allow for the calculation of Quality-Adjusted Life Years (QALYs) for use in the economic evaluation of health care interventions for this age group. However, such value sets are not currently available; therefore, a level sum score (LSS), like that used in the EQ-5D, was used to describe the responses on the descriptive system. Level labels are treated as numeric data with the best possible score as (1 + 1 + 1 + 1 + 1 + 1) = 6 and the most severe score as (3 + 3 + 3 + 3 + 3 + 3) = 18 [19]. The EQ-TIPS was designed to be amenable to developing preference weights in the future.

The final part of the EQ-TIPS, the EQ VAS, is used to record the proxy’s view of how good or bad the young child’s health is overall on the day of questionnaire completion. The EQ VAS is a vertical visual analogue scale with endpoints of 0 and 100 labelled “The worst health you can imagine” and “The best health you can imagine”, respectively. The standardised EQ VAS is adopted from the EQ-5D and EQ-5D-Y instruments [6].

### 2.3. Procedure

Ethical approval for pooled analyses was obtained from the University Human Research and Ethics Committee (HREC Ref: 740_2020). The details on ethics approval, consent, and procedure can be found in the corresponding publications [9,10,11,12,13].

### 2.4. Data Management and Analysis

Participants were grouped according to age groups based on their birthdate. The EQ-TIPS sample characteristics and item responses were summarised in terms of the frequency of responses to each item across the age categories and for the total sample and compared with the Kruskal–Wallis H. Similarly, the Median and Inter Quartile Range (IQR) of the EQ-TIPS LSS and VAS scores were calculated across age groups and by recruitment setting (inpatient and outpatient) and compared with Kruskal–Wallis H and pairwise comparison of age groups with Bonferroni correction for multiple tests or Mann–Whitney U Test. Spearman’s r was used to explore the association between EQ-TIPS items by age group and for the total sample. The correlation coefficients were interpreted according to Cohen: 0.1–0.29 low association, 0.3–0.49 moderate association, and ≥0.5 high association [20].

## 3. Results

In total, 551 infants/toddlers aged between 0 and 48 months were included. There were more toddlers in the 12–24-month group (n = 147, 27%) compared to the other age groups (range 17–20%). Although there were more males in total and within each age group, except 24–36 months, this was not significantly different (*p* = 0.194) (Table 1). There were, however, significantly more infants/toddlers recruited from the inpatient hospital setting in the 0–6-month group compared to the 24–36-month group (*p* = 0.012). Unsurprisingly, most caregivers who responded to the EQ-TIPS were mothers across all age groups (*p* = 0.726) (Table 1).

The health condition was categorised according to the outpatient clinic they were recruited from or the presenting condition for inpatient care. A maximum of two conditions were reported; however, an overlap was noted, e.g., some had a primary neurodevelopmental condition, whilst for others this was a second diagnosis. Thus, aggregated developmental delay ranged between 20 and 25% across the age groups. The categorised conditions were wide-ranging in their presentation, e.g., respiratory concerns ranged from chronic genetic conditions (cystic fibrosis) to acute infections with pneumonia and pulmonary TB. The category of other was wide-ranging and included metabolic disease, dermatology, ophthalmology, feeding difficulties, gastroenteritis, and acute bacterial and viral infections. 

**Figure 1:**
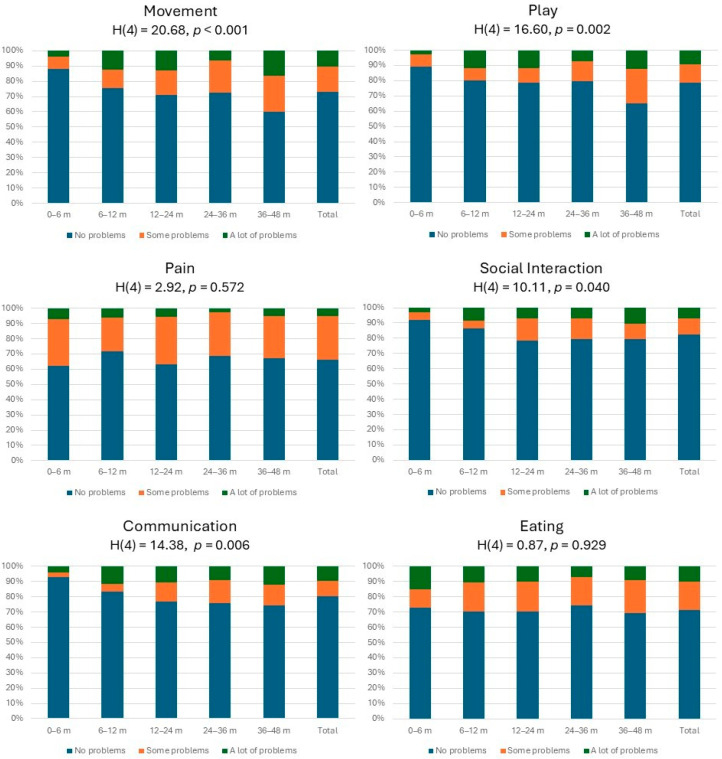
Proportion of EQ-TIPS problems by age group and total.

Figure 1 shows the distribution of responses by EQ-TIPS items by age group. The items of pain and eating showed no differences in responses across age groups. For the items of movement, play, and communication, there were significantly higher reports of no problem in the 0–6-month group compared to the 36–48-month group. Furthermore, for items of movement and communication, there was a significantly higher report of no problems in the 0–6-month group compared to the 12–24-month group. Considering the total report of no problems, this was highest for social interaction and communication. The report of ceiling effect (111111) was similar across age groups (F = 3.32, *p* = 0.506) and was 39% for the total sample. The floor effect (333333) was only reported for two children across the sample.

Table 2 compares the EQ-TIPS LSS and VAS scores across age groups and by recruitment setting (inpatient and outpatient). The LSS showed no significant differences by age group for the total sample. In contrast, the VAS score was significantly higher (indicating better general health) in the 24–36-month group when compared to the 36–48-month group (*p* = 0.015). When these scores were compared for those recruited from inpatient and outpatient care, there were significant differences noted for the LSS for the 12–24-month and 24–36-month groups. However, the VAS scores were significantly higher (indicating better general health) for the 0–6-month, 12–24-month, and 24–36-month groups.

The association between items by age group and total is shown in Table 3. Pain was not highly associated with any other item. Eating had no clear pattern of association with other items by age group, and total associations were low-to-moderate (range 0.22–0.37). Play was highly associated with movement, communication, and social interaction (excluding the 0–6-month group). Similarly, communication was highly associated with social interaction across the age groups and total. The 6–12-month group had the greatest number of highly associated items, whereas the 0–6-month group had the lowest.

## 4. Discussion

The results of this study highlight that the performance of the EQ-TIPS is related to both the age of the infant/toddler and the current overlap with item concepts. Although the age of infant/toddler may influence the conceptual overlap of the items, this discussion aims to highlight the most relevant concern.

Infants/toddlers were recruited from a paediatric tertiary level hospital and their diagnoses were thus complex in nature, making the categorisation thereof challenging. Consequently, attributing any changes in the performance of the item to the health condition was limited. The heterogenous and complex nature of the conditions was, however, likely to be equally distributed across the age groups and is unlikely to have influenced the reports. The importance of co-morbid conditions, their influence on the infant/toddlers functioning, and the disparity between healthy peers may, however, become more notable with age. This was not captured in this dataset and could be studied in the future in groups of children with more heterogenous health conditions.

The best differentiation of health conditions was perhaps the categorisation of those seeking inpatient or outpatient care. The pattern of health care utilisation in this sample indicates that there was a higher use of inpatient care in the youngest, most vulnerable, 0–6-month group when compared to the older age groups. Children with congenital or neonatal diagnoses of chronic conditions are further likely to be receiving necessary intervention in the acute setting for improved long-term outcomes, e.g., cardiac surgery, ventricular–peritoneal shunts, spinal closures, tracheostomy insertion, alternative feeding routes, surgical correction, etc. This may, however, be limited, as many children could have an acute or chronic presentation. This information was not recorded. Older toddlers, most notably the 36–48-month group, showed a higher use of outpatient or chronic health care services. Although the significantly higher report of problems (EQ-TIPS LSS) and worse general health (EQ VAS) cannot be directly attributed to the higher use of outpatient care, it does indicate age-related differences in reporting in this age group. These differences were most notable in the items linked to developmental milestones (movement, play, and communication ). This may be attributed to the fact that these items make a comparison to age-appropriate behaviour, and it is within this age group where deviation from peers becomes most apparent. For children with health conditions associated with delayed developmental milestones, their dependence on care may not have been considered problematic until they reach the age of 36–48 months, when they typically enter preschool and develop relative independence from their caregivers [21]. It is anticipated that, across the age trajectory into older childhood, many more problems will become apparent with neuromaturation, e.g., coordination, perception, attention, and learning [22].

Neuromaturation may be further driving the fact that the 6–12-month group had the greatest number of highly associated items, whereas the 0–6-month group had the lowest. Typically, infants have a small repertoire of skills in the first 6 months and usually spend a large amount of time feeding and sleeping therefore limiting the caregivers’ ability to detect problems and judge the severity thereof [23,24,25]. Infants reach a greater number of readily observable milestones between 6 and 12 months, which typically include eating solids, sitting, crawling, vocalising first words, and more animated and intentional interaction. These milestones most often develop interdependently [25]. High associations with eating and movement/play were notable in the 6–12-month age. Eating, and the emergence of self-feeding, in this age group is strongly linked to motor ability, including but not limited to the ability to sit, maintain adequate head control, and manipulate food in hands and hands to mouth [26]. Given that self-feeding at this age is primarily exploratory, its association with play is to be expected [27]. Unsurprisingly, as eating skills mature with age, this association naturally diminishes.

The item content for pain and eating appears to be appropriate with no age-related differences in performance and no or few associations with other items. The strong association between items of play and movement, communication, and play, communication, and social interaction across all ages indicates overlap in item content. This overlap could be due to an overlap in skills needed to perform functions in this age group and/or due to the redundancy of items. The association between play and movement is not unlike that previously reported between the EQ-5D-Y dimensions of Mobility and Usual Activities (for example, going to school, hobbies, sports, playing or doing things with family and friends) [28] or between items of Physical Functioning on the PedsQL and Usual Activities on the EQ-5D-Y-3L [11,28,29]. These associations with play in the infant and toddler period are, however, more nuanced, as play is well recognised as the vehicle by which the significant development of physical, cognitive, emotional, and social skills advances in this period [30,31,32]. Active play, or physical activity in a playful manner, is generally recognised during early infancy and toddlerhood where they use their emerging physical abilities to explore the environment [31,32]. It is argued that the manipulation of objects, symbolising play, problem solving, pretend play, and interactive play, contributes to the development of complex mental structures governing socio-emotional and socio-cognitive skills [30,32], although play in the EQ-TIPS refers to objects and toys that caregivers innately consider a larger definition of play. To reduce subjectivity, a wider definition of play should be considered for the EQ-TIPS. Although there is overlap between the constructs of play, movement, and communication, as play is considered the main occupation of a child [33], its retention may be warranted. Further research with caregivers is warranted to understand its relevance and whether there is concern about this overlap.

The association between communication and social interaction may be more inextricably linked. Clinically, these are distinct but related concepts; this may, however, not be apparent to the caregiver completing the EQ-TIPS. Social interaction was originally included in the EQ-TIPS as an item to capture emotional functioning; this has since been poorly correlated to items of emotion included on the PedsQL [11]. It was, however, strongly associated with items related to personal social development and communication on the Ages and Stages Questionnaire [9]. This may indicate that the item of social interaction, as currently worded, is interpreted as verbal and non-verbal interaction and playful interaction with familiar people. As currently worded, this is likely to make either the items of social interaction or communication redundant. The Diagnostic Classification of Mental Health and Development Disorders of Infancy and Early Childhood refers to behaviours of social communication, which includes verbal and non-verbal communications and interactions during social contexts [21]. In many instances, this may be further linked to social–emotional development and responsivity [21]. Considering the impact on HRQoL [34] and objective judgement by the caregiver, or another proxy respondent, redefining the items on the EQ-TIPS to represent an item of social communication and/or (social) emotion may be warranted. Although communication was identified as an important concept for inclusion during the initial development of the EQ-TIPS [8], this item may be a better measure of development than as an indicator of the impact of poor health on functioning and subsequently HRQoL.

The HSCS-PS was assessed for concurrent validity by comparing to the Bayley Scales for Infant development; the Vineland Adaptive Behaviour Scale and the Stanford–Binet total scores to individual HSCS-PS items, and the correlations between individual constructs were not assessed. There were no further age-related analyses [17]. Similarly, the PedsQL infant scales were compared to the Kessler-6 psychological distress scale for summary scores and total scores only [35]. Hadley-Smith et al. reported a small correlation between PedsQL scores and age, and although not significant, there was an indication that children older than 2 years had a clear separation between EQ VAS and PedsQL scores [36]. These results indicate that further investigation of the age-related performance of the PedsQL may be warranted. To date, there is no available evidence on the concurrent validity nor of the age-specific performance of the IQI.

This study was limited by the existing datasets, which did not all contain comparator measures of HRQoL and/or clinical measures to define known groups. Further analysis comparing psychometric performance across age groups could provide further evidence of item performance. These results could not further provide any evidence of performance against other measures or indicate whether there were any missing items/concepts in the EQ-TIPS. A further limiting factor was that socio-demographic information about the caregivers including their socioeconomic and educational status or their caregiving experience (e.g., number of children) was not collected. This may have impacted the reporting of the EQ-TIPS.

## 5. Conclusions

The results of this study highlight that the performance of the EQ-TIPS is related to both the age of the infant/toddler and the current overlap with item concepts. The significantly higher report of problems (EQ-TIPS LSS) and worse general health (VAS) cannot be directly attributed to the higher incidence of chronic health conditions. It does indicate that there is an age-related difference in the reporting of items linked to developmental milestones including movement, play, and communication. It is postulated that these deficits are more notable in children aged 36–48 months due to both deviation in skills when compared to peers and where dependence on caregivers usually decreases. The item content for pain and eating appears to be appropriate with no age-related differences in performance and no or few associations with other items. The strong association between items of play and movement, communication, and play, communication, and social interaction across all ages indicates overlap in item content. Although caregivers seem to have a wider reference of abilities when considering the item of play, it may be warranted to include broader examples in the EQ-TIPS. The overlap between communication and social interaction indicates redundancy. Redefining the items on the EQ-TIPS to represent an item of social communication and/or (social) emotion may be warranted. Future research should explore the psychometric performance of items, particularly the performance against other measures, to further inform item inclusion and revision, or whether there are any missing concepts.

## Figures and Tables

**Table 1:** Descriptive characteristics by age group and total.

n (%)			0–6 Months	6–12 Months	12–24 Months	24–36 Months	36–48 Months	Total
			n = 100	n = 95	n = 147	n = 112	n = 97	n = 551
Sex	Male		56 (56%)	57 (60%)	86 (59%)	54 (48%)	52 (54%)	305 (55%)
Recruitment Setting	Inpatient		64 (64%)	52 (55%)	60 (41%)	37 (33%)	46 (47%)	259 (47%)
	Outpatient		36 (36%)	42 (44%)	58 (39%)	54 (48%)	51 (53%)	241 (44%)
Caregiver Relationship to Child	Mother		96 (96%)	86 (91%)	131 (89%)	100 (89%)	85 (88%)	498 (90%)
	Father		2 (2%)	3 (3%)	4 (3%)	4 (4%)	7 (7%)	20 (4%)
	Grandparent		2 (2%)	4 (4%)	6 (4%)	4 (4%)	3 (3%)	19 (3%)
	Other (Aunt, Uncle, Stepmother, Sister, Foster Mother)		0	2 (2%)	6 (4%)	4 (4%)	2 (2%)	14 (3%)
Health Condition	Neurodevelopmental	Total	14 (14%)	20 (21%)	30 (20%)	19 (17%)	21 (22%)	104 (19%)
		CP	3 (3%)	3 (3%)	7 (5%)	10 (9%)	13 (13%)	36 (7%)
		Genetic Syndrome	3 (3%)	7 (7%)	6 (4%)	2 (2%)	1 (1%)	19 (3%)
		Structural Brain Abnormality	3 (3%)	2 (2%)	4 (3%)	2 (2%)	0	11 (2%)
		Developmental Delay	0	1 (1%)	4 (3%)	0	1 (1%)	6 (1%)
		Epilepsy	0	2 (2%)	1 (1%)	2 (2%)	0	5 (1%)
		Meningitis	0	1 (1%)	0	0	2 (2%)	3 (1%)
		Neuromuscular Disease	0	0	0	0	3 (3%)	3 (1%)
		Spina Bifida	3 (3%)	0	1 (1%)	0	0	4 (1%)
		Other	2 (2%)	4 (4%)	7 (5%)	3 (3%)	1 (1%)	17 (3%)
	Respiratory	Total	16 (16%)	13 (14%)	17 (12%)	15 (13%)	19 (20%)	80 (15%)
		Atopy (Allergy/Asthma/Eczema)	1 (1%)	5 (5%)	9 (6%)	5 (4%)	8 (8%)	28 (5%)
		Cystic Fibrosis	4 (4%)	3 (3%)	1 (1%)	4 (4%)	5 (5%)	17 (3%)
		Pneumonia	4 (4%)	2 (2%)	2 (1%)	0	3 (3%)	11 (2%)
		Pulmonary TB	3 (3%)	0	0	1 (1%)	1 (1%)	5 (1%)
		Other	4 (4%)	3 (3%)	5 (3%)	5 (4%)	2 (2%)	19 (3%)
	Tracheostomy (+/−ventilation)	Total	13 (13%)	4 (4%)	12 (8%)	13 (12%)	6 (6%)	48 (9%)
		Neurodevelopmental	9 (9%)	1 (1%)	7 (5%)	9 (8%)	1 (1%)	27 (5%)
		UAO	4 (4%)	3 (3%)	5 (3%)	4 (4%)	5 (5%)	21 (4%)
	General Surgery	Total	15 (15%)	11 (11%)	13 (11%)	10 (13%)	8 (8%)	57 (10%)
	Burn	Total	1 (1%)	9 (9%)	16 (11%)	15 (13%)	11 (11%)	52 (9%)
	Congenital Cardiac Disease	Total	18 (18%)	9 (9%)	7 (5%)	7 (6%)	2 (2%)	43 (8%)
	GIT	Total	4 (4%)	10 (10%)	4 (3%)	5 (4%)	4 (4%)	27 (5%)
	Orthopaedic	Total	0	1 (1%)	0	4 (4%)	6 (6%)	11 (2%)
	Other	Total	18 (18%)	17 (18%)	44 (30%)	17 (15%)	10 (10%)	106 (19%)

CP: Cerebral Palsy, TB: Tuberculosis, UAO: Upper Airway Obstruction, GIT: Gastro-intestinal tract disease.

**Table 2:** EQ-TIPS level sum score and visual analogue scale score by age group and recruitment setting.

			0–6 Months	6–12 Months	12–24 Months	24–36 Months	36–48 Months	Total
			n = 100	n = 95	n = 147	n = 112	n = 97	n = 551
EQ-TIPS LSS	Total	Median (IQR)	7 (6, 8)	7 (6, 8)	7 (6, 8)	7 (6, 9)	8 (6, 10)	7 (6, 8)
		Kruskal–Wallis H (*p*-value)	H (4) = 8.60 (*p* = 0.072)					
	Inpatient	n	64	52	60	37	46	259
		Median (IQR)	7 (6, 8)	7 (6, 9)	8 (7, 10)	8 (6, 9)	7 (6, 9)	7 (6, 9)
	Outpatient	n	36	42	58	54	51	241
		Median (IQR)	7 (6, 8)	7 (6, 8)	7 (6, 8)	7 (6, 8)	8 (6, 10)	7 (6, 8)
		Mann–Whitney U (*p*-value)	1486 (*p* = 0.225)	1500 (*p* = 0.309)	3718 (*p* < 0.001)	2608 (*p* = 0.013)	2173 (*p* = 0.623)	33,587 (*p* = 0.127)
EQ-TIPS VAS	Total	Median (IQR)	85(65, 100)	80 (60, 100)	90 (70, 100)	90 (70, 100)	80 (55, 91)	85 (65, 100)
		Kruskal–Wallis H (*p*-value)	H (4) = 12.59 (*p* = 0.013)					
	Inpatient	n	64	52	60	37	46	259
		Median (IQR)	80 (55, 95)	80 (50, 98)	80 (50, 95)	75 (60, 100)	80 (55, 95)	80 (50, 95)
	Outpatient	n	36	42	58	54	51	241
		Median (IQR)	90 (80, 100)	80 (70, 100)	91 (72, 100)	95 (80, 100)	80 (50, 90)	90 (70, 100)
		Mann–Whitney U (*p*-value)	941 (*p* = 0.018)	1115 (*p* = 0.118)	1962 (*p* = 0.0)	1462 (*p* = 0.006)	1958 (*p* = 0.677)	25,634 (*p* < 0.001)

LSS: level sum score; a higher score indicates worse HRQoL. VAS: visual analogue scale; a higher score indicates better general health.

**Table 3:** Association between EQ-TIPS items by age group and total.

		0–6 Months	6–12 Months	12–24 Months	24–36 Months	36–48 Months	Total
		n = 100	n = 95	n = 147	n = 112	n = 97	n = 551
Movement	Play	0.60 **	0.77 **	0.68 **	0.39 **	0.71 **	0.65 **
	Pain	0.18	0.08	0.19 *	0.27 *	0.26 *	0.20 **
	Social Interaction	0.14	0.53 **	0.31 **	0.39 **	0.46 **	0.39 **
	Communication	0.40 **	0.69 **	0.66 **	0.36 **	0.51 **	0.55 **
	Eating	0.36 **	0.53 **	0.25 **	0.17	0.18	0.28 **
Play	Pain	0.26 *	0.07	0.25 *	0.13	0.34 *	0.20 **
	Social Interaction	0.27 *	0.62 **	0.54 **	0.64 **	0.59 **	0.56 **
	Communication	0.42 **	0.67 **	0.79 **	0.53 **	0.61 **	0.64 **
	Eating	0.31 **	0.54 **	0.32 **	0.31 **	0.26 *	0.34 **
Pain	Social Interaction	0.01	0.11	0.16	0.12	0.25 *	0.13 *
	Communication	0.05	0.04	0.16 *	0.08	0.18 *	0.10 *
	Eating	0.15	0.17	0.35 **	0.30 *	0.31 *	0.26 **
Social Interaction	Communication	0.51 **	0.73 **	0.56 **	0.58 **	0.69 **	0.62 **
	Eating	0.24 *	0.42 **	0.38 **	0.43 **	0.23 *	0.34 **
Communication	Eating	0.32 **	0.44 **	0.25 **	0.23 **	0.20	0.27 **

* Correlation is significant *p* < 0.05 (2-tailed). ** Correlation is significant *p* < 0.001 (2-tailed). Shading indicates high correlations > 0.50.

## Data Availability

The datasets presented in this article are not readily available, as development of the EQ-TIPS and data analyses are ongoing. Requests to access the datasets should be directed to janine.verstraete@uct.ac.za. The EQ-TIPS is an experimental version; permission for use needs to be obtained from the EuroQol Research Foundation.
